# Reconstructing Articular Cartilage in the *Australopithecus afarensis* Hip Joint and the Need for Modeling Six Degrees of Freedom

**DOI:** 10.1093/iob/obac031

**Published:** 2022-07-28

**Authors:** Ashleigh L A Wiseman, Oliver E Demuth, Emma Pomeroy, Isabelle De Groote

**Affiliations:** McDonald Institute for Archaeological Research, University of Cambridge, Cambridge CB2 1TN; Research Centre in Evolutionary Anthropology and Paleoecology, Liverpool John Moores University, Liverpool, Merseyside L3 5UX; Department of Earth Sciences, University of Cambridge, Cambridge CB2 1TN; Structure and Motion Laboratory, Royal Veterinary College, London NW1 0TU; Department of Archaeology, University of Cambridge, Cambridge CB2 1TN; Department of Archaeology, Ghent University, 9000 Ghent

## Abstract

The postcranial skeleton of *Australopithecus afarensis* (AL 288–1) exhibits clear adaptations for bipedality, although there is some debate as to the efficiency and frequency of such upright movement. Some researchers argue that AL 288–1 walked with an erect limb like modern humans do, whilst others advocate for a “bent-hip bent-knee” (BHBK) gait, although in recent years the general consensus favors erect bipedalism. To date, no quantitative method has addressed the articulation of the AL 288–1 hip joint, nor its range of motion (ROM) with consideration for joint spacing, used as a proxy for the thickness of the articular cartilage present within the joint spacing which can affect how a joint moves. Here, we employed ROM mapping methods to estimate the joint spacing of AL 288–1’s hip joint in comparison to a modern human and chimpanzee. Nine simulations assessed different joint spacing and tested the range of joint congruency (i.e., ranging from a closely packed socket to loosely packed). We further evaluated the sphericity of the femoral head and whether three rotational degrees of freedom (DOFs) sufficiently captures the full ROM or if translational DOFs must be included. With both setups, we found that the AL 288–1 hip was unlikely to be highly congruent (as it is in modern humans) because this would severely restrict hip rotational movement and would severely limit the capability for both bipedality and even arboreal locomotion. Rather, the hip was more cartilaginous than it is in the modern humans, permitting the hip to rotate into positions necessitated by both terrestrial and arboreal movements. Rotational-only simulations found that AL 288–1 was unable to extend the hip like modern humans, forcing the specimen to employ a BHBK style of walking, thus contradicting 40+ years of previous research into the locomotory capabilities of AL 288–1. Therefore, we advocate that differences in the sphericity of the AL 288–1 femoral head with that of a modern human necessitates all six DOFs to be included in which AL 288–1 could osteologically extend the hip to facilitate a human-like gait.

## German

Das postkraniale Skelett von *Australopithecus afarensis* (AL 288–1) weisst deutliche Anpassungen für einen aufrechten Gang auf, allerdings ist die Effizienz und Häufigkeit der aufrechten Fortbewegung umstritten. Einige Forscher argumentieren, dass AL 288–1 wie der moderne Mensch mit aufrechten Beinen lief, während andere der Ansicht sind einen Gang mit gebeugter Hüfte und gebeugtem Knie (BHBK—*bent-hip bent-knee*) scheine wahrscheinlicher, obwohl in den letzten Jahren der allgemeine Konsens die aufrechte Zweibeinigkeit bevorzugt. Bislang wurde mit quantitativen Methoden weder die Artikulation des AL 288–1 Hüftgelenkes noch dessen Bewegungsumfang (ROM—*range of motion*), mit Einbezug des Gelenkzwischenraumes untersucht, welcher als Indikator für die Dicke des Gelenkknorpels dient und beeinflussen kann, wie sich ein Gelenk bewegt. Hier verwendeten wir die ROM-Mapping-Methode, um den Abstand zwischen den Gelenksflächen in der Hüfte von AL 288–1, im Vergleich zu einem modernen Menschen und einem Schimpansen, zu bestimmen. In neun Simulationen wurden unterschiedliche Gelenkabstände und die Variationsbreite der Gelenkskongruenz (d. h. von einer enganliegenden bis zu einer locker sitzenden Hüftpfanne) getestet. Darüber hinaus haben wir die Kugelförmigkeit des Oberschenkelkopfes untersucht und geprüft, ob drei rotierende Freiheitsgrade (DOFs—*degrees of freedom*) ausreichen, um das gesamte ROM zu erfassen, oder ob zusätzlich translatorische DOFs einbezogen werden müssen. Bei beiden Versuchsanordnungen stellten wir fest, dass die Hüfte von AL 288–1 wahrscheinlich nicht hochgradig kongruent war (im Gegensatz zum modernen Menschen), da dies die Rotationsfähigkeit der Hüfte stark eingeschränkt und sowohl die Fähigkeit zur zweibeinigen Fortbewegung als auch zu deren in den Bäumen stark beeinträchtigt hätte. Stattdessen war die Hüfte vermutlich mit mehr Knorpel ausgestattet als beim modernen Menschen, so dass sie sich in Positionen drehen konnte, die sowohl für Bewegungen am Boden als auch in den Bäumen erforderlich waren. Reine Rotations-Simulationen ergaben, dass AL 288–1 nicht in der Lage gewesen wäre, die Hüfte wie ein moderner Mensch zu strecken, so dass das Individuum gezwungen gewesen wäre, im BHBK-Stil zu gehen, was mehr als 40 Jahre vorangegangener Forschung über die Fortbewegungsfähigkeiten von AL 288–1 widersprochen hätte. Daher sind wir der Auffassung, dass aufgrund der Unterschiede in der Kugelförmigkeit des Hüftkopfes von AL 288–1 und demjenigen eines modernen Menschen alle sechs DOFs einbezogen werden müssen, damit AL 288–1 die Hüfte osteologisch so strecken konnte, dass ein menschenähnlicher Gang möglich gewesen wäre.

## Dutch

Het postcraniële skelet van *Australopithecus afarensis* (AL 288–1) vertoont duidelijke aanpassingen voor tweevoetigheid, hoewel er enige discussie bestaat over de efficiëntie en de frequentie van deze rechtopgaande beweging. Sommige onderzoekers beweren dat AL 288–1 met een recht ledemaat liep zoals de moderne mens, terwijl anderen pleiten voor een “bent-hip bent-knee” (BHBK (vertaling: *gebogen-heup gebogen-knie*) gang, hoewel de laatste jaren de algemene consensus uitgaat naar een erect bipedalisme. Tot op heden heeft geen enkele kwantitatieve methode zich gebogen over de articulatie van het AL 288–1 heupgewricht, noch over het bewegingsbereik (ROM) met inachtneming van de gewrichtsafstand, die wordt gebruikt als benadering voor de dikte van het gewrichtskraakbeen dat in de gewrichtsafstand aanwezig is en dat de manier waarop een gewricht beweegt kan beïnvloeden. Hier hebben we ROM mapping methoden gebruikt om de kraakbeendikte en gewrichtsafstand van het heupgewricht van AL 288–1 te schatten in vergelijking met die van een moderne mens en chimpansee. Negen simulaties evalueerden verschillende gewrichtsafstanden en testten het bereik van gewrichtscongruentie (d.w.z., variërend van een dicht opeen gepakte kom tot een los opeengepakte kom). Verder evalueerden wij de bolvormigheid van de femurkop en of drie roterende vrijheidsgraden (DOF's—*degrees of freedom*) de volledige ROM voldoende weergeven of dat de translatorische DOF's moeten worden meegenomen. In beide gevallen bleek dat de heup van de AL 288–1 waarschijnlijk niet zeer congruent was (zoals wordt gezien bij de moderne mens), omdat dit de rotatiebeweging van de heup ernstig zou beperken en het vermogen tot tweevoetigheid en zelfs tot voortbewegen in bomen ernstig zou beperken. In plaats daarvan had de heup meer kraakbeen dan bij de moderne mens, waardoor de heup kon roteren in posities die nodig zijn voor zowel bewegingen op het land als in de bomen. Simulaties van enkel rotatie toonden aan dat AL 288–1 niet in staat was om de heup te strekken zoals de moderne mens, waardoor het specimen gedwongen werd om een BHBK manier van lopen toe te passen. Dit is in tegenspraak met meer dan 40 jaar van eerder onderzoek naar de locomotorische capaciteiten van AL 288–1. Daarom stellen wij dat de verschillen in bolvorm van de femurkop van AL 288–1 ten opzichte van die van de moderne mens vereisen dat alle zes DOFs worden opgenomen waarin AL 288–1 de heup osteologisch zou kunnen strekken om een mensachtige loopbeweging mogelijk te maken.

## Introduction

Limb movement is a fundamental question in evolutionary studies and in recent years osteological range of motion (ROM) mapping methods have been developed for a select range of extinct and extant species to ascertain how two body segments articulate and move relative to one another (Pierce et al. 2012; Nyakatura et al. 2015; Manafzadeh & Padian 2018; Demuth et al. 2020; Manafzadeh & Gatesy 2021; Manafzadeh et al. 2021; Richards et al. 2021). ROM mapping relies upon movement of a body segment relative to another around a joint center and can encompass rotation and/or translational movement (Manafzadeh & Padian 2018; Demuth et al. 2020; Manafzadeh & Gatesy 2021). The method identifies which poses are viable and which are non-viable based on bone morphology, thus providing information regarding habitual limb posture, such as distinguishing a biped from a quadruped (Pierce et al. 2012; Demuth et al. 2020; Brocklehurst et al. 2021).

To date, this method has not yet been applied to humans nor the hominin fossil record, but offers potential to address questions regarding the bipedal gait of early hominins, such as the probable biped *Australopithecus afarensis* (Gruss et al. 2017). The specimen AL 288–1 (commonly known as “Lucy”) is one of the most complete hominin specimens, dated to 3.2 million years ago (Ma) from the Hadar region of Ethiopia (Johanson et al. 1982; Kimbel et al. 1994). Researchers generally agree that the postcranial skeleton displays morphological features indicative of bipedality (Rak 1991; Kramer 1999; Ward 2002; Wang et al. 2004; Lovejoy 2005; Nagano et al. 2005; Gruss et al. 2017), despite numerous morphological differences to modern humans, such as a wide pelvis and relatively shorter lower limbs (Jungers 1982; McHenry 1986; Tague & Lovejoy 1986; Kramer 1999; Wall-Scheffler & Myers 2017). These and other skeletal differences (Brassey et al. 2018) have underpinned two schools of thought: *Au. afarensis* as a facultative biped that exploited other locomotory avenues such as arborealism (Senut 1980; Stern 2000) versus *Au. afarensis* as a habitual biped, mostly exploiting bipedalism as the main form of locomotion (Preuschoft & Witte 1991; Rak 1991; Lovejoy 2007). These arguments have been further bolstered by the discovery of the Laetoli footprints, dated to 3.66 Ma and attributed to *Au. afarensis* (Leakey and Hay 1979; Masao et al. 2016), from which arguments regarding limb posture were (and somewhat remain) quite polarized, with researchers arguing over whether the track-makers walked with an extended limb or a bent hip-bent knee (BHBK) posture (Day and Wickens 1980; White and Suwa 1987; Tuttle et al. 1990; Sellers et al. 2005; Berge et al. 2006; Raichlen et al. 2008; Tuttle 2008; Raichlen et al. 2010; Meldrum et al. 2011; Crompton et al. 2012; Hatala et al. 2016).

Reconstructing anatomical possibilities for the ROM of key joints in the body which directly influence how a species moves (i.e., the hip) may help to resolve these debates. ROM mapping (Manafzadeh & Padian 2018; Manafzadeh & Gatesy 2021, 2022) offers the potential to quantitatively measure the digital articulation of the hip joint and to ascertain if certain limb poses which were essential for bipedality were osteologically possible or not—that is, could AL 288–1 position their hip joint in flexed and extended rotations required by bipedal poses to permit forward movement? Or were these poses osteologically restricted, thus prohibiting an extended limb posture and possibly indicating that a BHBK bipedal gait must have been employed?

Consideration must also be given to articular cartilage/joint spacing (henceforth, articular cartilage will be referred to as just “cartilage”). Unfortunately, cartilage does not usually preserve in the fossil record (Holliday et al. 2010; Mallison 2010) and so is not present in the AL 288–1 specimen. Yet, every synovial joint in the living adult tetrapod body contains viscoelastic cartilage which is a smooth surface that acts to dissipate mechanical stress, upon which two body segments move relative to one another (Fox et al. 2009). No differences in cartilage thickness have been previously identified between bipeds and quadrupeds (Bonnan et al. 2013), but differences do reflect mammalian body size, whereby larger mammals have relatively thinner cartilage than smaller mammals (Stockwell 1971; Biewener 2005; Bonnan et al. 2013; Malda et al. 2013), and this relationship typically scales with negative allometry (Malda et al. 2013), but see (Simon 1971). Adult mammals with larger body sizes typically have more congruent joints (i.e., thinner cartilage), in which stress is dissipated into the underlying subchondral bone via more closely articulated joint surfaces rather than via the cartilage itself (Simon 1970; Simon 1971; Simon et al. 1973; Fuss 1994). Smaller adult mammals, on the other hand, typically have less congruent joints and, as a result, have relatively thicker cartilage for stress dissipation (Fuss 1994; Bonnan et al. 2013). Here, “congruence” is defined as when two opposing articulating surfaces are most similar and packed tightly together, although the range of congruence across species scales with body mass (Simon et al. 1973).

The estimated body mass of AL 288–1 ranges from 13 to 42 kg (Johanson & Edey 1981; McHenry 1992; Ruff 2010; Grabowski et al. 2015; Brassey et al. 2018), whereas body masses of modern human adults are typically greater than this (∼50–100 + kg), although with much temporal and ecogeographical variation between and within populations (Ruff 2002). Chimpanzees (*Pan troglodytes*) from the Gombe National Park, Tanzania have a median body mass of 39 kg (males) and 31.3 kg (females) (Pusey et al. 2005). Therefore, it is logical to assume that the smaller AL 288–1 specimen had proportionally thicker cartilage than modern humans and potentially chimpanzees (if using the lower range of body mass estimates of AL 288–1) based upon scaling assumptions (Bonnan et al. 2013; Malda et al. 2013).

**Fig. 1:**
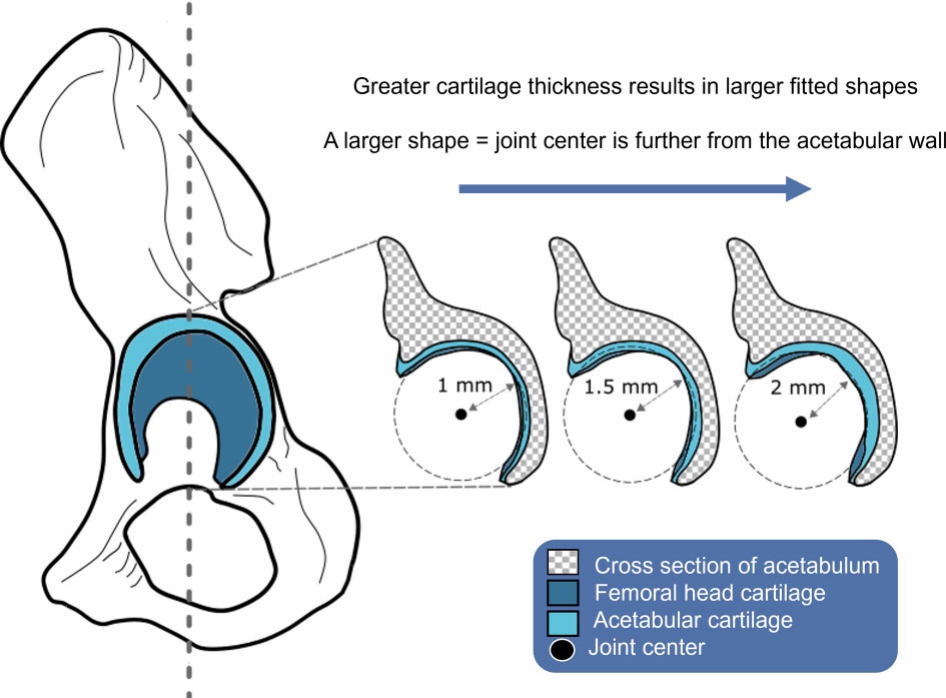
Hypothetical diagram explaining how cartilage thickness in the hip joint affects the establishment of the joint center position. The shape of the acetabulum and femoral head are used to create fitted shapes, as shown here as figurative dashed circles (not to scale). The centroids of each created shape are then superimposed from which the shape centers are used to determine the joint center for disarticulated specimens. If a specimen has a greater amount of cartilage, then the fitted shape is larger, thus moving the joint center away from the acetabulum. All movement occurs around this joint center, meaning that, theoretically for the AL 288–1 hip joint, a joint center which is not tightly constrained inside the hip permits greater rotational movement.

Cartilage thickness also varies within the hip joint itself (Kurrat & Oberlander 1978; Shepherd and Seedhom 1999). Kurrat and Oberlander (Kurrat & Oberlander 1978) established three important patterns regarding the distribution of cartilage thickness: (1) maximum thickness in the acetabulum is located in the ventrocranial region, whereas in the femoral head this is ventrolaterally positioned; (2) cartilage thickness typically decreases concentrically towards the borders of the cartilage rims; and (3) in the resting position of the hip joint (i.e., during standing with the femur perpendicular to the ground), the thickest regions do not align. Consequently, modeling cartilage thickness may seem problematic (Tsai et al. 2020). Fortunately, shape fitting procedures which are used to establish joint centers (Kambic et al. 2014; Bishop et al. 2020; Wiseman et al. 2021; Gatesy et al. 2022) and also rearticulate disarticulated skeletal elements (Bishop et al. 2020; Demuth et al. 2020) take into consideration the full averaged shape of the articulating surfaces, and so circumvent the need to model differing cartilage distributions by providing a standardized thickness throughout the joint. From the shape of the articular surface, a primitive shape (e.g., a sphere) is fitted (Bishop et al. 2020; Gatesy et al. 2022) which approximates and represents the averaged articulating surface, and is, therefore, suited for reconstructing joint centers in fossil taxa (Demuth et al. 2020). Assuming that the shape of the overlaying cartilage in AL 288–1 was the same as the underlying subchondral bone shape (i.e., the cartilage has the same thickness relative to the bone articular shape), then the articular surface shape will be accurate (Bonnan et al. 2010), but the joint spacing might be wrong without understanding the thickness of the cartilage itself (Fig. 1).

The 3D joint center position depends on the thickness of the cartilage (Taylor & Wedel 2013; Vidal et al. 2020), whereby thinner cartilage will produce a joint center which is more representative of a highly congruent joint and thicker cartilage will produce a joint center which is located further from the acetabular wall (Fig. 1). The positioning of the joint center is fundamental in establishing rotational and translational movement—from this center (which is located inside the femoral head), all movement occurs. Therefore, we must accurately reconstruct cartilage and determine its thickness in the AL 288–1 hip joint to gain insights into locomotor modes by establishing if cartilage thickness scales as expected (i.e., by being less congruent than a larger-bodied analogous species).

Finally, we must consider how a joint articulates and moves to be able to comprehensively reconstruct cartilage thickness. A joint is capable of rotational movement, which—in *Animalia*—is represented as movement along three rotational axes/degrees of freedom (DOF): flexion-extension (FE), abduction-adduction, and long axis rotation (LAR) (Manafzadeh et al. 2021). However, it has been well-established that the true ROM of a joint is only accurately reconstructed by also including translational DOFs (Manafzadeh & Gatesy 2021). Joints which are highly osteologically constrained, such as mammalian hip joints, are thought to not require all DOFs to be modeled due to (1) the high congruency of the joint and (2) the sphericity of the femoral head and acetabulum. As such, it could be argued that the AL 288–1 hip's ROM is sufficiently quantified by only modeling three rotational DOFs. However, this remains to be tested on a greater variety of species and translational DOFs should not be ignored at risk of excluding viable poses (Manafzadeh & Gatesy 2021). If translational DOFs were excluded, we might, therefore, make broad and possibly incorrect assumptions regarding the functional capabilities of AL 288–1’s hip by not capturing the full range of mobility. It might also be possible to sufficiently capture a joint's ROM by modeling four DOFs which would consist of three rotational DOFs (dynamic) and a singular translational DOF (static), following (Demuth et al. 2020). Such approach would involve systematically increasing joint spacing along one axis (moving the distal element further away from the joint center) and then computing rotational movement around the proximal joint center location.

Furthermore, such modeling of joint ROM would be redundant without comparison to empirical motion data. Assuming that *Au. afarensis* was bipedal, it is likely that this species walked and ran barefoot on unstable and rugged terrain, such as those found on the African savannah (Winder et al. 2015). We need to explore limb function over similar terrain to fully understand the evolutionary pressures experienced by the lower limb. Only by doing so, we will understand the ranges of motion required by a joint to facilitate realistic movement.

Joint spacing was used as a proxy for cartilage thickness. Here, we modeled differing joint spacings in the AL 288–1 hip joint based upon measurements from extant *Pan troglodytes* and *Homo sapiens* in two different ways. First, we used the four DOF approach (three dynamic rotations with one static translation) to ascertain if this could estimate cartilage thickness by methodically increasing the joint spacing based upon scaling assumptions. Second, we included six DOFs to determine if all DOFs are truly needed to accurately estimate hip mobility, thus addressing the question of if estimates of cartilage thickness alongside modeling assumptions affect predictions of how AL 288–1 walked. Such investigations have important palaeobiological implications for studies which neglect to model translational movement in fossils.

## Materials and methods

### Specimen acquisition

We used the reconstructed AL 288–1 (*Au. afarensis*) lower limb model by Brassey and colleagues (2018). Only the pelvis and left femur of this previous reconstruction were included here. Whilst we acknowledge that there might be potential reconstruction issues in the rotation of the os-coxae in this reconstruction, this is negligible for the current study in which only the left femur and left ilium/acetabulum will influence results. This was confirmed by a sensitivity analysis in which we digitally manipulated the femur around the joint center and confirmed that the femur does not collide with any other bony element of the pelvis without first colliding with the acetabular wall. Future studies using this pelvis for other research goals should consider reconstructive modifications to the sacrum and flaring aspects of the ilia.

For the comparative modern specimens we sourced high-quality, open-access CT data of lower limb models from which 3D models were readily available. This resulted in one modern, male human (*Homo sapiens*) lower limb model obtained from (Modenese & Renault 2021) and one modern, male chimpanzee (specimen ID: YPM MAM 015,939; *P. troglodytes*) lower limb model which was provided by the Yale Peabody Museum of Natural History (obtained from MorphoSource).

The modern human specimen used in this study weighed 84 kg (Modenese & Renault 2021), which is a body mass discrepancy of ∼50–84% between AL 288–1 and the modern human based upon old body mass estimates, although this is likely to be ∼76% based upon recent convex hull approaches of AL 288–1 (Brassey et al. 2018). The chimpanzee specimen had a body mass of 36.3 kg, which is ∼44% larger than AL 288–1 based upon this more recent AL 288–1 convex hull approach.

### Simulation setup

**Fig. 2:**
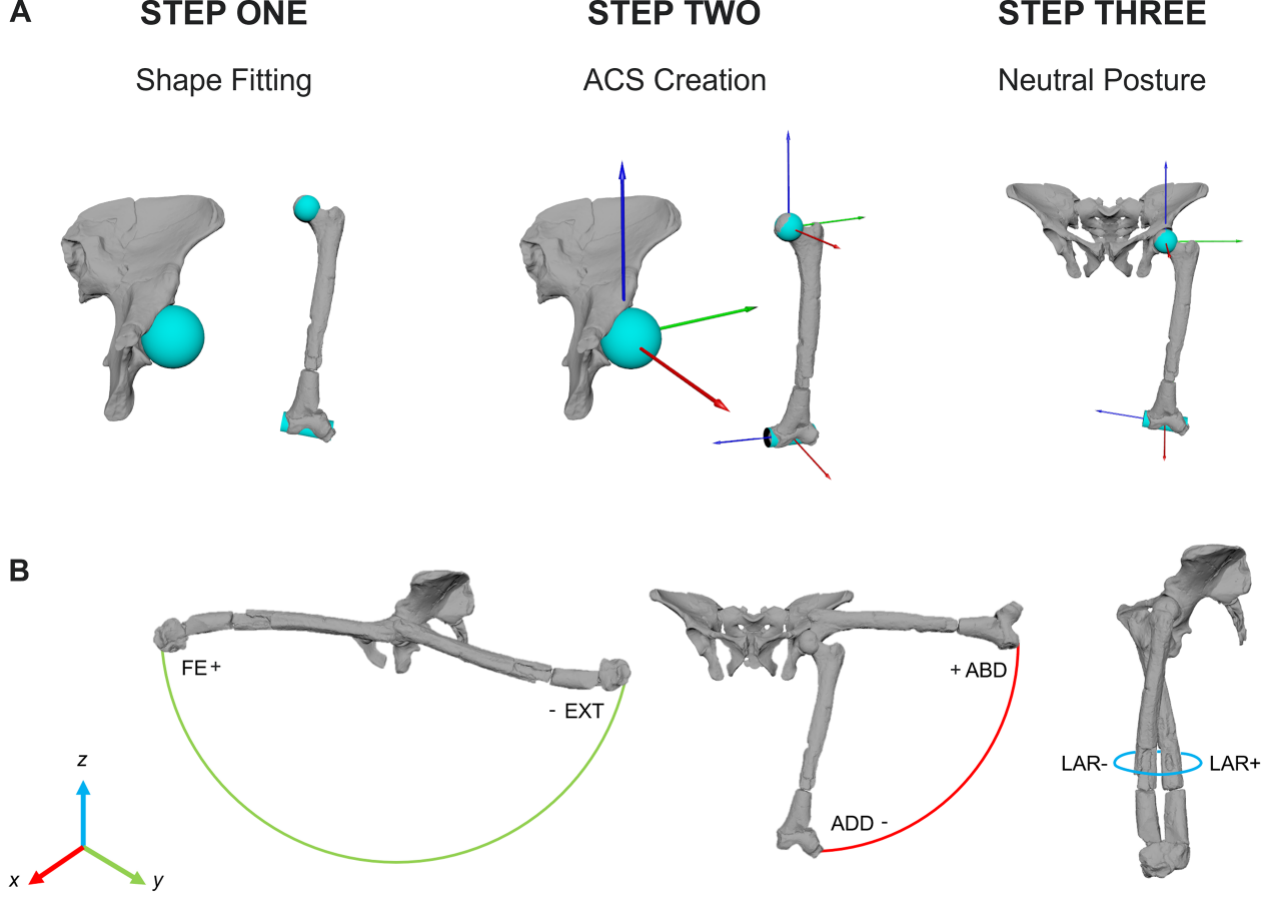
(A) Step by step process of the initial setup. Step one involves the shape fitting procedure, which also assists in the rearticulation of the bones. Step two involves the ACS creation. Step three is the neutral posture setup, which forms the starting pose. From the starting pose, all joint angles are set to 0 and joint positioning then deviates from this “0” pose. (B) Examples of the three degrees of freedom used in this study: flexion-extension (FE/EXT), adduction-abduction (ADD/ABD), and long axis rotation (LAR; or external (+) and internal (−) rotation). Rotation was only modeled around the hip joint in this study.

The workflow for this study involves the following steps (Fig. 2):

1. Shape fitting procedure.
2. Anatomical coordinate system (ACS) creation.
  - Re-articulation for AL 288–1.
3. Neutral posture setup.
4. Modeling joint mobility.

For step one, a shape fitting procedure was applied to all specimens (Kambic et al. 2014; Bishop et al. 2020; Demuth et al. 2020; Wiseman et al. 2021; Gatesy et al. 2022). In brief, spheres were fitted to each acetabulum and to the femoral head, and cylinders were fitted to the sacrum and femoral condyles. These shapes were used to create and align the ACSs for the pelvic center, left hip and left knee joint in which polylines connecting each shape's centroid directed the formation of each axis (for further details in shape-fitting refer to (Bishop et al. 2020; Gatesy et al. 2022)). Prior to ACS creation, it was first necessary to rearticulate the AL 288–1 specimen. Rearticulation of AL 288–1 involved overlaying the centroids of the fitted primitives of the acetabulum and femoral head which directly mapped the sphericity of these elements (Otero et al. 2017; Klinkhamer et al. 2018; Bishop et al. 2020; Demuth et al. 2020). No re-articulation of the human or chimpanzee models was required because these specimens were CT-scanned in articulation.

ACSs were established for the pelvis, left hip joint and the left knee joint (Fig. 2a) (Kambic et al. 2014, 2017; Gatesy et al. 2022). The hip joint was permitted three rotational DOF. The X axis was abduction (+ rotation)/adduction (− rotation), Y was flexion (+ rotation)/extension (− rotation), and Z was long-axis rotation (+ external rotation;—internal rotation), with the coordinate system as shown in Fig. 2a and implemented in Maya 2022 (Autodesk Inc., San Rafael, USA), following (Manafzadeh & Padian 2018) with a XYZ rotation order within Maya. Rotation order was selected to be comparable with the previously collected empirical motion data (see: Section 2.5). Two types of simulation were created: (1) rotational-only with a singular static Y-axis translation to systematically test increasing joint spacing (Demuth et al. 2020); and (2) rotational and dynamic translational DOFs were included. Both types were included to test if the sphericity of the Hominini femoral head superseded the requirement to model six DOFs (note: here, Hominini refers to the tribe comprising humans, chimpanzees, bonobos, and their fossil ancestors; hominin refers to just humans and their fossil relatives). For example, during comparisons of *ex-vivo* kinematic data of osteological ROM analyses, Manafzadeh and Gatesy (2021) determined that translational movement in the joints substantially influences joint mobility and thus affect the resultant ROM maps. Due to the sphericity of the Hominini (human, chimpanzee and AL 288–1) hip joint which would theoretically constrain the femoral head during movement, there is the possibility that the joint center might be accurately established using only the overlaid fitted spheres and, therefore, rendering translational DOFs as negligible.

Each modeled specimen was set up in the “neutral posture” (step three; Fig. 2a), which is used here as our “starting position,” although in other studies these poses may differ (Kambic et al. 2014; Bishop et al. 2020; Gatesy et al. 2022). In this pose, all joint angles were set to 0°,0°,0° (Kambic et al. 2014) from which all rotational movement deviates, with the joint axes permitting movement along each DOF. Importantly, the neutral posture has no influence on the resultant movement and does not necessarily have to reflect any realistic anatomical position (Kambic et al. 2014; Gatesy et al. 2022). Rather, all movement deviates from this neutral posture, allowing the study and data to be comparable between subjects and reusable by future researchers in which the researcher will know precisely how to position the pose 90°,45°,60° (example pose) from this starting position, assuming the rotation order is consistent.

The neutral posture was established using a forward kinematic rig (Kambic et al. 2014; Manafzadeh & Padian 2018; Demuth et al. 2020), with the femur extending towards the ground whereby the Z-axis was perpendicular to the ground. Because this study only focused on the hip joint, it was not necessary to create ACSs for any other lower limb joint and the knee ACS was discarded after neutral posture setup.

### Measuring cartilage thickness in comparative specimens

To determine joint spacing in the AL 288–1 hip, it was necessary to set up several simulations in which cartilage thickness varied. Cartilage thickness is not a “one size fits all” per species, with individuals having different thicknesses, albeit within a species range (Kurrat & Oberlander 1978). Therefore, we sought to model a range of thicknesses according to the chimpanzee and human comparative samples. Due to limited cadaveric specimens, all chimpanzee measurements were obtained from the same specimen. We measured cartilage thickness at 15 different sections of the chimpanzee hip joint from the raw CT-stack in the coronal plane (specimen ID: YPM MAM 015,939) using ImageJ (Doube et al. 2010). From these measurements, we extracted the minimum, average, and maximum spacing of the joint (see Table 1), and then scaled these values by femoral length to the AL 288–1 specimen as a proxy for applying the chimpanzee range of thickness to AL 288–1. All joint spacing values represent the maximum spacing which may be found within a joint, owing to cartilage thickness varying within a joint itself (Kurrat & Oberlander 1978). We do not provide a minimum-maximum range here.

**Table 1:** List of *A. afarensis* simulations computed in this study, including an overview of the thickness of the AC modeled. Note that the three DOF simulation (no single axis translation included) of the overlaid fitted primitives produced no viable poses whatsoever. All reported cartilage thicknesses for AL 288–1 are scaled by femoral length (289 mm as measured from the reconstructed model) (Brassey et al. 2018), with the percentage of cartilage to femoral length in *Au. afarensis* reported. AL 288–1 simulation numbers are sequentially ordered with respect to cartilage thickness, with simulation 1 being the smallest modeled thickness and simulation 6 being the greatest.

Specimen	Simulation	AC thickness	AC as % of femur length	Source of AC thickness/Justification
***Four DOF Simulations***				
AL 288–1	0	–	–	Overlaid primitives; simulation produced 0 viable poses
AL 288–1	1	0.764 mm	0.264%	Minimum human thickness
AL 288–1	2	1.183 mm	0.409%	Minimum chimpanzee thickness
AL 288–1	3	1.951 mm	0.675%	Average human thickness
AL 288–1	4	2.587 mm	0.895%	Maximum human thickness
AL 288–1	5	3.069 mm	1.062%	Average chimpanzee thickness
AL 288–1	6	5.321 mm	1.841%	Maximum chimpanzee thickness
Modern human	7	CT-scanned in articulation	–	–
Modern chimpanzee	8	CT-scanned in articulation	–	–
***Six DOF Simulation***				
AL 288–1	9	Radius = 2.448 mm	–	Overlaid primitives

Unfortunately, the 3D bone geometries of the human specimen were triangulated and smoothed (Modenese & Renault 2021), so it could not be reliably ascertained if the cartilage was precisely represented, other than an estimation of the joint centers. Therefore, we used published values of human cartilage thicknesses from four individuals (Kurrat & Oberlander 1978) to accurately establish the minimum, average, and maximum thickness in the human hip, which were then scaled by femoral length to AL 288–1. To validate the 3D human model/simulation, we measured the joint spacing from the 3D model (*n* = 1) at 15 different intervals and determined that the joint spacing range (0.267–0.903% of femoral length) fell within the range of the published values from the four individuals (Kurrat & Oberlander 1978; Mechlenburg et al. 2007) (0.262–1.046% of femoral length), which was further promising for validating the chimpanzee measurements where *n* = 1.

For AL 288–1, six forward kinematic rigs were setup, each with different joint spacing. Joint spacing was altered via single-axis translation along the Y-axis, following (Demuth et al. 2020; Manafzadeh & Gatesy 2021). In theory, these simulations may be referred to as four DOFs in which we permitted a static single-axis translation per simulation, alongside three dynamic, rotational DOFs. Henceforth, these simulations will be referred to as “four DOF.”

Previously it has been argued that the difference in the radii of the fitted shapes approximates joint spacing in extant species (Crocodylians) and thus may also be representative for some extinct species (Demuth et al. 2020), but due to a lack of cartilage preservation in most extinct species this remains an uncertainty, necessitating the four DOF versus six DOF approach used here. A seventh AL 288–1 rig was established which permitted three rotational and three translational DOFs using the same XYZ rotation order. We adapted the prism-style method of translation developed by Manafzadeh and Gatesy (2021) for hinge joints by using a sphere rather than a prism, which was composed of 32 faces (axis division of 8; height division of 4). The radius of the sphere was set to the difference between the radii of the fitted shapes of the acetabulum and femoral head (2.448 mm), thus ensuring that the magnitude of the translational offset remained constant, which was a necessary adaptation of this previous approach for a ball and socket joint. During the simulation, the ACS moved to each of the vertices of the sphere and sampled rotational poses. This sphere-approach is in contrast to (Manafzadeh & Gatesy 2021) in which a prism-style approach was instead implemented for a hinge-type joint whereby the corners of the cube acted as reference points for the ACS from which the motion is applied. The individual lengths of the cube's axes in this previous study were measured via *ex vivo* experimentation. However, if the maximal offsets are directly applied to a ball and socket joint, then the cube's corners might be out with realistic maximal translational capabilities and would possibly reflect a disarticulated joint and, consequently, might sample too many poses. The sphere-approach used here ensures that these offsets are constrained within the sphericity of the acetabulum and do not become disarticulated (MEL code provided in Supplementary Information 2).

A single six DOF simulation was computed for AL 288–1 which used the difference of the radii of the fitted shapes of the acetabulum and femoral head as the maximal amount of translation in each direction. Because cartilage does not preserve in the fossil record, palaeontological studies typically use the difference in radii of the acetabulum and femoral head or a percentage of the long bone lengths as the basis for joint spacing in the specimen (Demuth et al. 2020), thereby implying that the difference in radii should sufficiently capture the ROM in four DOF simulations (here, Simulation 3; Table 1). By adding in translational movement (here, Simulation 9; Table 1), additional sampling occurs which will capture a greater range of poses by accurately reflecting the dynamic motion of a joint. By including Simulation 9, we are directly testing if we need translational DOFs to be included to fully represent the ROM of the AL 288–1 hip joint, or if four DOFs are sufficient and can be used to ascertain the likelihood of cartilage thickness and the resultant osteological mobility, as can be accomplished for extinct archosaurs (Demuth et al. 2020).

For comparability with the *Au. afarensis* data, human and chimpanzee simulations used the four DOF approach. Due to the fact that the extant specimens were scanned with their joints in articulation, the joint centers could be accurately estimated and, additionally, the full spectrum of osteological poses was already captured solely in the four DOF simulations (i.e., no missing sections of mobility are present, rather a full envelope of motion as captured, rendering the four DOF simulations sufficient; see Results), it was not necessary to model six DOFs in the human (Simulation 7) or chimpanzee (Simulation 8) specimens.

### Simulations

A 3D joint sampling approach (Manafzadeh & Padian 2018; Demuth et al. 2020; Richards et al. 2021; Manafzadeh & Gatesy 2022) was implemented to estimate the 3D ROM of each specimen's hip. A total of nine simulations were set up (Table 1), of which six modeled various joint spacings via a single axis translation in the AL 288–1 hip. The FE and LAR axes were both permitted to move through a range of −180° to 180°. Abduction-adduction (ABAD) had a range of −90° to 90°. The joint was then rotated into 197,173 possible poses for all simulations following (Manafzadeh & Padian 2018; Demuth et al. 2020). Modified *Maya Embedded Language* (MEL) code for the creation of six DOF simulations can be found in Supplementary Information 2. For each simulation, all possible joint rotation combinations were sampled at 5°-intervals and all non-viable poses (bone mesh interpenetration) were discarded (Manafzadeh & Padian 2018). An Euler cosine-corrected shape space (Manafzadeh & Gatesy 2021) was implemented in MATLAB 2021b to map the viable poses and produce an alpha shape (i.e., a solid volume of mapped degrees (°) which is a shape formed from the outer layer of 3D coordinates that represents each viable pose) of each simulation's ROM.

Assessment of our results was conducted in two parts, as follows:

1. We qualitatively compared all mapped rotational-only ROMs (the four DOF approach; Simulations 1–8) between each of the AL 288–1 simulations in comparison to the modern human and chimpanzee simulations to (1) establish the effect of joint spacing on joint mobility and (2) to determine how joint spacing influences the range of viable limb poses and the implications this may have for reconstructing past movement. We computed regressions to determine the relationship between joint spacing and viable poses and with volume to establish if increased joint spacing scales linearly with movement capability (i.e., does greater joint spacing permit more mobility?).
2. We compared the six DOF AL 288–1 simulation with the four DOF-approach simulations to establish if the sphericity of the Hominini hip joint supersedes the need for six DOFs to be modeled, or if six DOFs are a necessary requirement to accurately model the AL 288–1 hip ROM.

### 3D motion data comparisons

Finally, we compared the simulated joint angles to previous research on modern human lower limb joint angles (Wiseman 2019; Wiseman & De Groote 2021). In this previous study, participants were recruited to move across three different substrates of varying compliancy (i.e., ranging from hard ground to a soft, deformable substrate) at a walk, a fast walk, and a jog. We used a 14-camera optoelectronic 3D motion capture system (250 Hz, Oqus Cameras, Qualysis AB, Gothenburg, Sweden) to capture kinematics (i.e., the hip joint angles) across each substrate via a reflective marker-set. Details regarding trackway construction and 3D motion capture of human movement across different substrates can be found in Supplementary Information 1. Ethical approval was granted by the Liverpool John Moores University Research Ethics Committee (REC: 16/NSP/041).

Resultant joint angles from the modern human experiments were compared to the AL 288–1 viable poses to ascertain if AL 288–1 could have traversed less compliant substrates at various speeds, thereby potentially addressing questions of habitual versus facultative bipedal movement. For example, if humans required greater degrees of extension in the hip to walk and/or jog across a soft, easily deformable substrate (similar to the natrocarbonite ash from Laetoli, Tanzania) (Leakey and Hay 1979), do we see this osteological capability in AL 288–1 to facilitate such human-like movement and how might this functionally relate to cartilage thickness, in which joint spacing is used as a proxy?

## Results

### Results of the four DOF-approach simulations

**Fig. 3:**
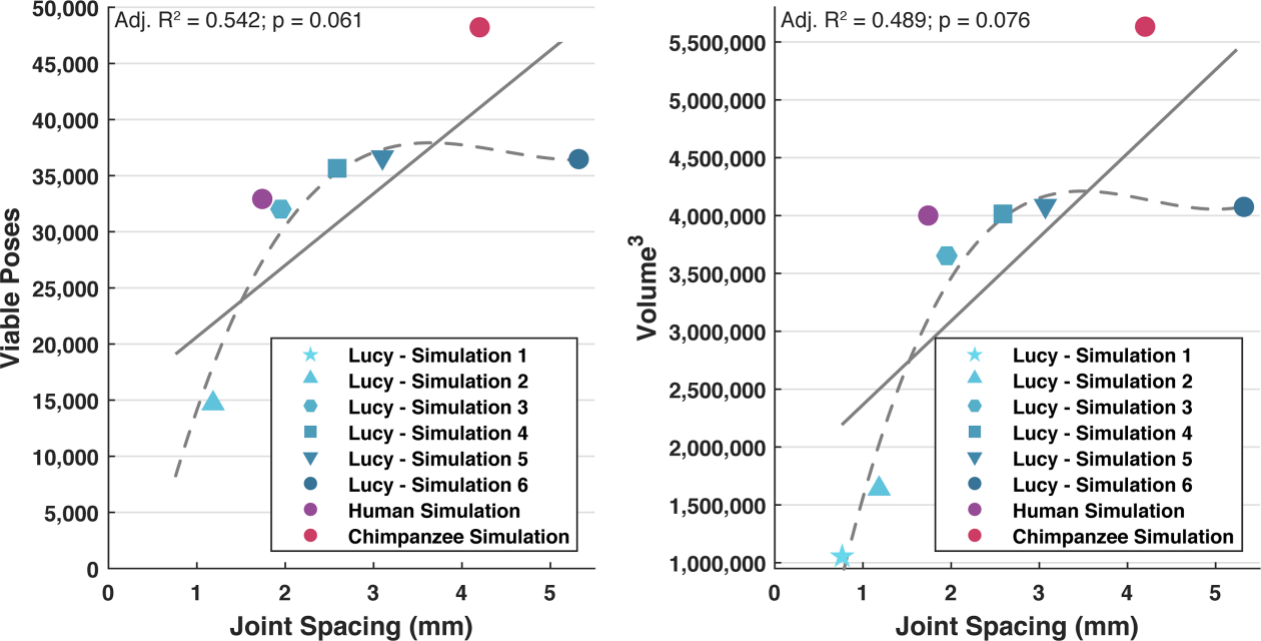
Relationship of joint spacing (mm) to the amount of viable poses produced (A) and to the volume 3 (B). Fitted regression lines are solid. Fitted polynomial lines to the AL 288–1 data points are dashed. The polynomial lines implies a plateau of the amount of viable poses/volume 3 possible, despite increasing joint spacing.

Results of the AL 288–1 simulations (simulations 1–6) indicated that those which had smaller joint spacing produced fewer viable poses and smaller volumes (degree^3^) (Table 2; Fig. 3). A greater volume defined by the amount of viable poses means that the hip joint will be more mobile, whereas a smaller volume/amount of viable poses means that the joint will be more restrictive. The chimpanzee produced the greatest amount of viable poses/volume, implying a highly mobile hip. AL 288–1 produced a range of 9,758 to 36,476 viable poses, with volume ranging from 1,048,249 degrees^3^ (restrictive) to 4,075,208 degrees^3^ (highly mobile). Simulations 1–2 produced ROMs that had substantially smaller volumes than other simulations and also far lower than the ranges of the chimpanzee (18.61% and 29.21%, respectively, of its volume) and modern human (26.20% and 41.13%, respectively, of its volume). This indicates that more congruent joints (simulations 1–2) permitted less rotational movement in the hip than a more cartilaginous joint (simulations 3–6).

**Table 2:** List of each of the four DOF-approach simulations conducted in this study in which joint spacing differed, resulting in various amounts of viable poses and volume of degrees. Volume calculated from the cosine corrected data points (Manafzadeh & Gatesy 2020). See also: Fig. 3.

	Simulation	Viable poses	Volume (Degree^3^)
*Au. afarensis*	1	9,758	1,048,249
	2	14,890	1,645,521
	3	32,021	3,652,600
	4	35,655	4,014,458
	5	36,345	4,062,729
	6	36,476	4,075,208
Modern human	7	32,924	4,000,521
Modern chimpanzee	8	48,218	5,632,700

A regression of viable poses and volume on joint spacing to shows a negative allometric relationship in both cases (*n* = 8 simulations). There was a weak correlation between joint spacing and viable poses (Adj. R^2^ = 0.542; *P* = 0.061) and volumes (Adj. R^2^ = 0.489; *P* = 0.076). However, if we consider just the AL 288–1 simulations (*n* = 6), there is a trend for both viable poses and volume to increase with increasing joint spacing, although this appears to plateau around ∼2–2.5 mm of joint spacing (as indicated by the dashed trendline in Fig. 3). Although we cautiously interpret these regressed results due to a small sample size (*n* = 6), joint mobility in the AL 288–1 hip joint does not increase as linearly as expected with joint spacing (i.e., it would be expected that as joint spacing increased, then so would mobility as the femoral head moved further from the acetabulum resulting in fewer bony collisions). This may indicate a maximum threshold for thickness above which greater joint spacing would have a negligible effect on greater rotational mobility. Importantly, this indicates that joint spacing in AL 288–1 scaled as predicted with the human and chimpanzee in which the hip was less congruent.

**Fig. 4:**
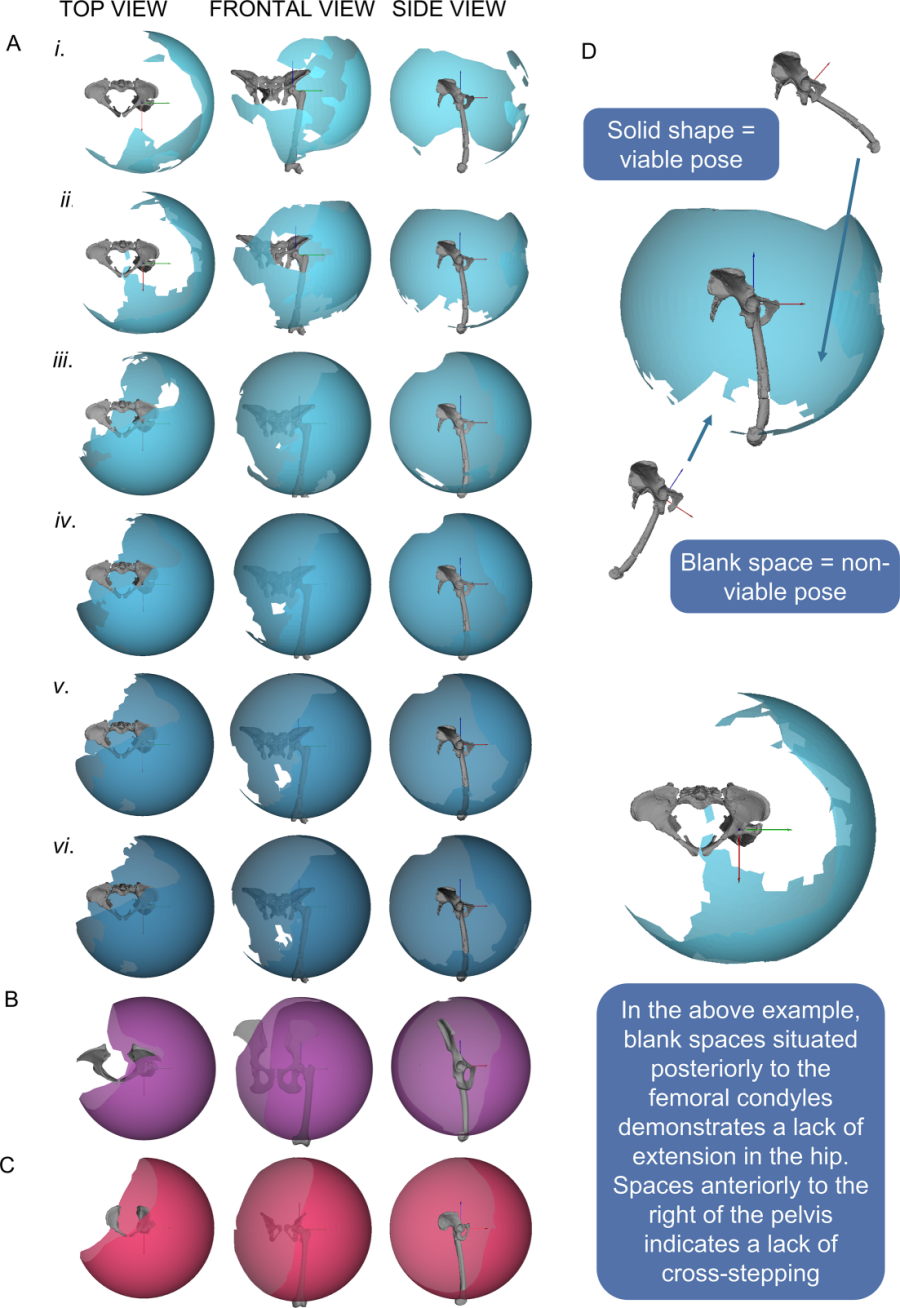
Results of the ROM mapping in which solid spheres representing all osteological viable poses to visualize how the femur can move in relation to the acetabulum. (A) The ROM maps for AL 288–1 with cartilage thickness of (i) 0.764 mm—simulation 1, (ii) 1.183 mm—simulation 2, (iii) 1.951 mm—simulation 3, (iv) 2.587 mm—simulation 4, (v) 3.069 mm—simulation 5, and (vi) 5.321 mm—simulation 6. Also shown, are the ROM maps for (B) a chimpanzee and (C) a modern human. (D) Annotated result with exemplar viable and unviable poses to help illustrate what the ROM maps represent. These ROM maps are solely osteologically constrained, that is, do not take into account additional constraints imposed by soft tissues surrounding the bones. Individual ROM maps can be found in Supplementary Information 3.

Osteological ROM maps are reported in Fig. 4. Whilst many of the reported poses such as those seen cranially to the pelvis are not used in habitual movement in extant species (and also likely extinct species too), such poses would likely be restricted by soft tissue constraints, not modeled here. We include the full envelope of osteological configurations to demonstrate how modeling different joint spacings drastically effects the resultant ROM map and inferred functionality of the joint.

Simulations 1–2 have a less dense map, with fewer viable poses (Fig. 4Ai, ii). As joint spacing is increased, the ROM maps become more dense with greater viable poses (Fig. 4Aiii-vi), which are similar to the chimpanzee and modern human simulations (Fig. 4B,100). Most unviable poses in simulations 4–8 are reflective of the body's inability to position the femur through the pelvis, and so the osteological envelopes in simulations 4–6 are likely “complete” (i.e., we would not expect additional viable poses to be generated if greater joint spacing was to be modeled) assuming only three DOFs are present (however, see below). This implies that the full breadth of realistic joint spacings were included.

**Fig. 5:**
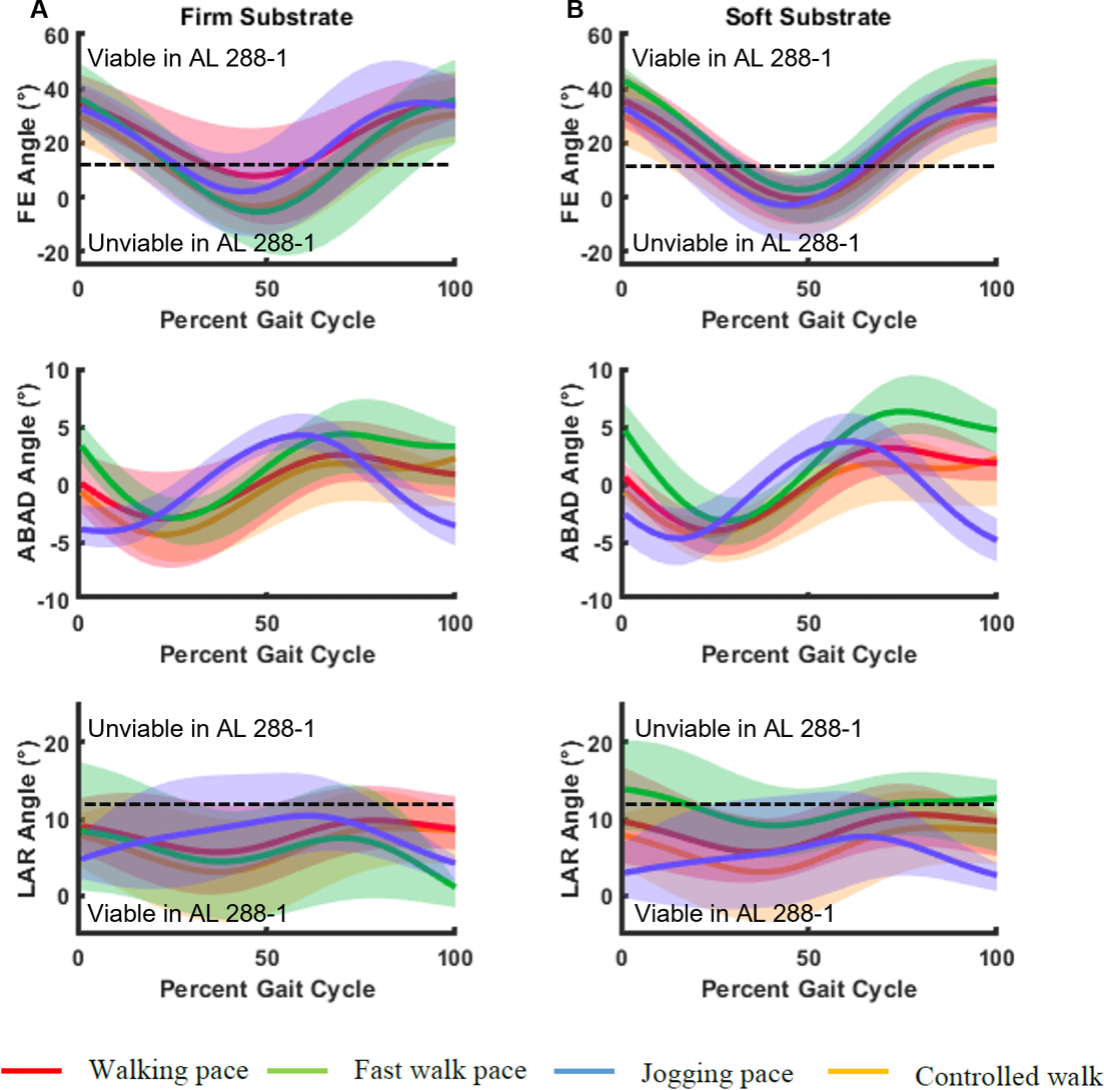
Results of the 3D motion capture of 40 humans walking across two different compliant substrates, a firm (A) and loose (B) substrate at various speeds. Joint angles were mostly consistent between each of the different walks. The dashed lines represent the division between viable and unviable poses in AL 288–1 in simulations 1–2. Rotation axes are the same as this study: flexion (+), extension (−), abduction (+), adduction (-), external rotation (+), and internal rotation (−).

If the joint was highly congruent (simulations 1–2), then AL 288–1 had limited mobility and an inability to extend the hip into positions necessitated by human-like bipedal walking across compliant substrates at various walking speeds, as indicated by the modern human experiments (Fig. 5). Therefore, we can infer that if AL 288–1 did have a highly congruent joint which isometrically scaled with modern humans (simulations 1–2), then AL 288–1 (1) was not a habitual biped, (2) was unlikely to be traversing a range of different substrates of varying compliancy, and (3) did not walk with an extended limb posture, and instead may have walked with a BHBK style of gait, similar to chimpanzees when they walk upright (O'Neill et al. 2015). However, simulations 1–2 only modeled a static translation, not dynamic translational movement of which will be discussed in the next section.

**Fig. 6:**
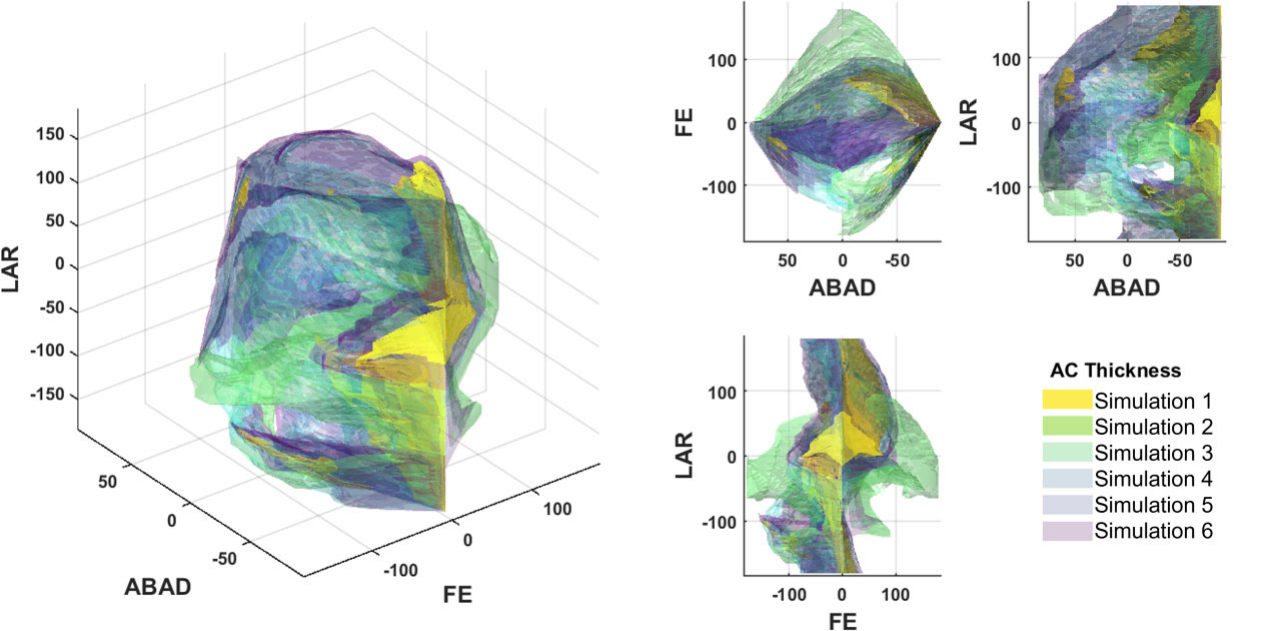
3D plots illustrating the shape differences between each of the alpha shapes representing the *Au. afarensis* ROM mapping. LAR = long axis rotation. ABAD = abduction/adduction. FE = flexion/extension. Results displayed here are cosine-corrected. Rotating 3D plots are available in Supplementary Information Video S1, with individual ROM figures available in SI3. Simulations 1 and 2 are not discussed in text due to unviability, but in brief: the alpha shapes are smaller, with many floating “islands.” It is impossible to reach such “islands” without being able to physically move the femur through the region of unviability, further discrediting the theory of high-congruence and dismissing simulations 1 and 2. Positive rotations correspond to a positive axis value; negative rotations correspond to a negative axis value.

Because too few poses are present in simulations 1–2 and ROM maps are similar between simulations 4–6, we can postulate that the likely maximum joint spacing must be a value somewhere between the spacings associated with simulations 3 (1.951 mm) to 6 (5.321 mm)—assuming that the sphericity of the hip joint elements supersedes dynamic translational modeling requirements. To test this, we explored the differences between each simulations’ alpha shape. This comparison highlights a few patterns (Fig. 6; Supplementary Video S1).

First, simulation 3 has greater flexion and extension than any other simulation (the lighter green alpha shape in Fig. 6; simulation 3), indicating that greater joint spacing (simulations 4–6) restricts the capability to flex and extend the hip. For example, as joint spacing increases in simulations 4–6, then the femoral head collides with the acetabular wall during hyper-flexed/extended positions producing unviable poses. However, this does not occur in simulation 3, whereby the femur can instead move through a greater range of movement before bony collision than all other simulations.

**Fig. 7:**
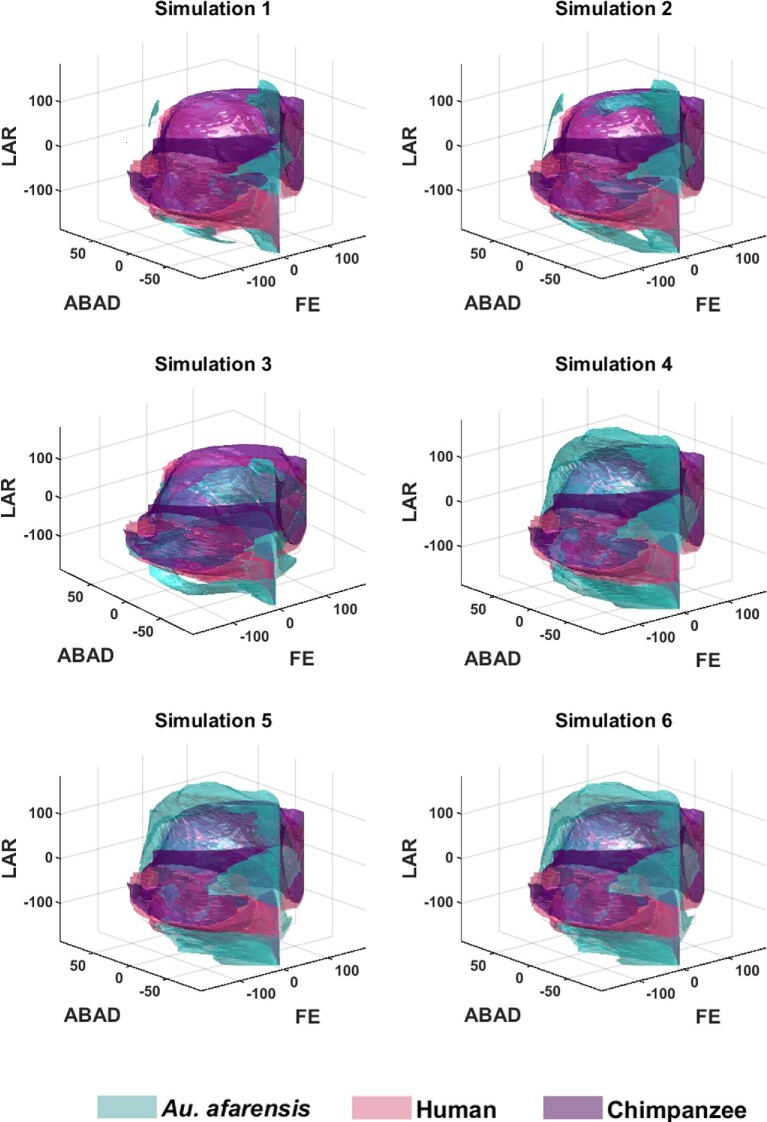
3D plots illustrating the shape differences between each of the alpha shapes from the AL-288–1 specimen (simulations 1–6; Table 1) in comparison to the modern human and chimpanzee specimens. LAR = long axis rotation. ABAD = abduction/adduction. FE = flexion/extension. Rotating 3D plots are available in Supplementary Information Videos S2. Positive rotations correspond to a positive axis value; negative rotations correspond to a negative axis value.

Second, simulations 4–6 have greater adduction and abduction (ABAD) than simulations 1–3 (e.g., the dark purple alpha shape in Fig. 6; simulation 6), indicating that greater joint spacing promotes ABAD in lieu of greater FE. Greater ABAD permits a greater range of posterior positioning of the hip, as indicated in the ROM maps in Fig. 4. Therefore, the ability to achieve greater abduction (positive rotations along the ABAD axis; Fig. 7) facilitates greater overall mobility if we consider all three rotational DOFs acting together to move the hip.

If we compare the AL 288–1 alpha shapes to those from the chimpanzee and human (Fig. 7; Supplementary Videos S2), we notice further patterns. First, simulations 1–6 all have greater LAR mobility than both the chimpanzee and human, in which the AL 288–1 alpha shapes (green alpha shape) demonstrates the ability to internally and externally rotate the hip more than the human (pink alpha shape) and chimpanzee (purple alpha shape) (Fig. 7). Second, simulations 1–6 have less flexion capability than the modern comparisons, but have greater extension of the hip in simulations 4–6. For example, the AL 288–1 alpha shapes (in turquoise; Fig. 7) in simulations 4–6 have broad similarity with the shapes of the human and chimpanzee along the flexion borders (flexion = negative angle). Importantly, simulations 1–6 all show that AL 288–1 had unique osteological mobilities, not present in the extant comparative specimens. AL 288–1 has > 105° greater rotation in the hip than a human and > 30° greater than a chimpanzee, present in abducted limb poses. This implies potential functional differences in limb mobility.

Overall, the results from simulations 1–6 demonstrates that a systematic increase in simulated joint spacing affects predictions of functionality.

### Results of the six DOF simulation

The six DOF simulation was computed using the starting point of the centroids of the overlaid primitives, in which translation (sliding of the joint) was permitted via movement around a sphere whereby the difference in radii represented the joint spacing. The three DOF simulation (no single axis translation included) of the overlaid primitives produced no viable poses due to constant mesh interpenetration. The six DOF simulation (simulation 9), on the other hand, produced 63,821 viable poses which far exceeded that of the human and chimpanzee ROMs (Table 2). It is likely that some of these poses would be restricted by soft tissue constraints (Manafzadeh & Padian 2018) which are not modeled here. The inclusion of dynamic translation in the simulation generated a greater spectrum of viability than those seen in simulations 1–6, but further inferences on limb mobility will only be possible by modeling ligamentous constraints in the future. For example, we cannot exclude the possibility that soft tissue restrictions might restrict this greater spectrum of poses indicated by the static simulations.

**Fig. 8:**
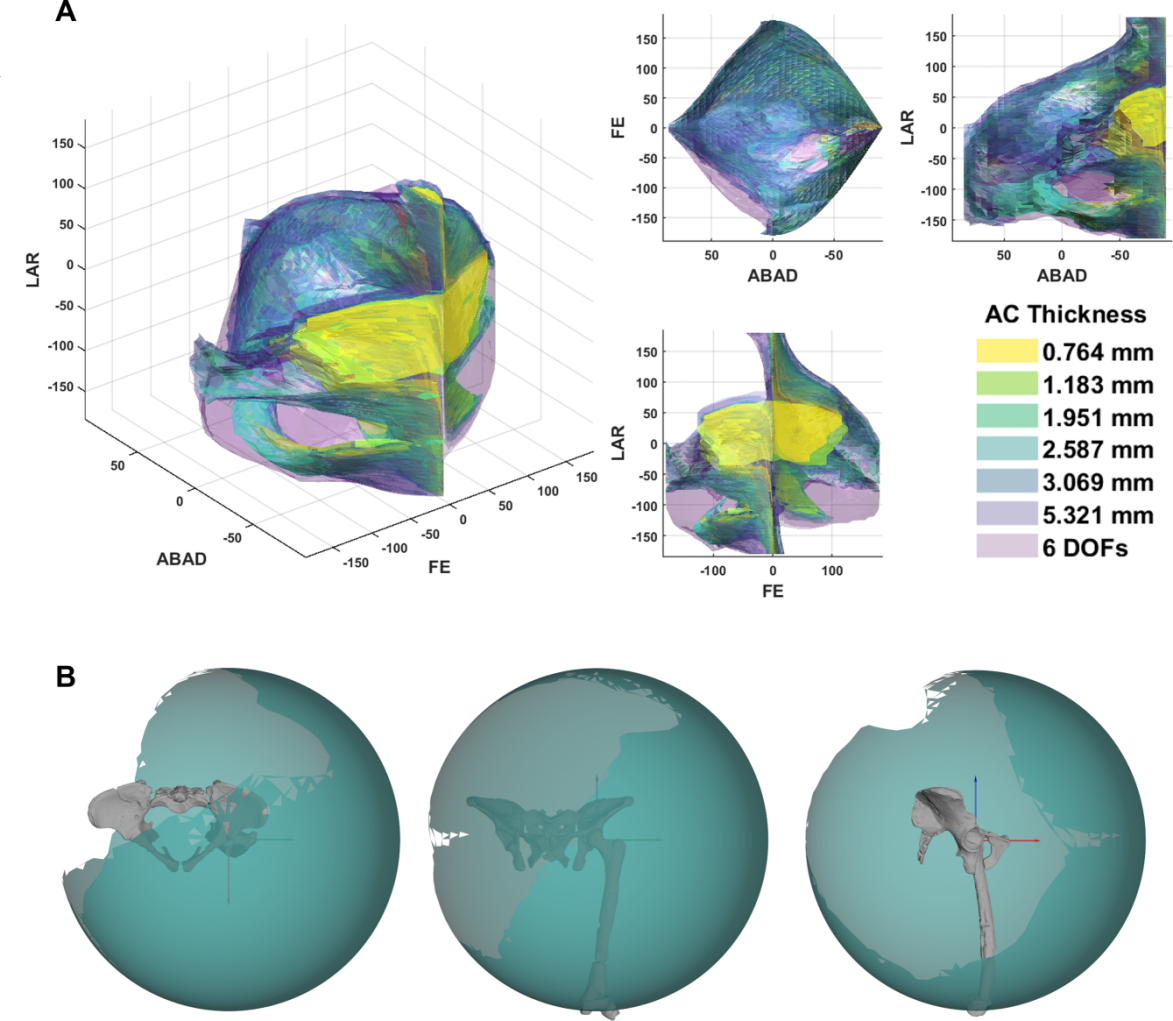
(A) 3D plots illustrating the shape differences between all AL 288–1 simulations, including the 6 DOF simulation. Results highlight many similarities in viability. A few differences are notable, such as increased ABAD poses not found in the other simulations. (B) Results of the ROM mapping from the 6 DOF simulation, in which the ROM map is complete in contrast to those shown in Fig. 4. Positive rotations correspond to a positive axis value; negative rotations correspond to a negative axis value.

In simulation 9, the hip was able to extend into positions necessitated by bipedal movement across a range of substrates at various speeds (Fig. 5), without the loss of hyper-flexion which was noted in simulations 3–6. Whilst we acknowledge that the alpha shapes of simulation 9 (Fig. 8) closely resembles those of simulations 3–6, there is a notable increase in the range of abducted/adducted poses in simulation 9 which is not present in the other AL 288–1 simulations. All these poses are osteologically feasible (i.e., the bones would not penetrate each other), but it is likely that some of these poses would be restricted by soft tissues.

## Discussion

In this study, we computed two assessments. The first modeled static translation of the hip joint center to determine if it is possible to predict joint spacing (a proxy for the maximum cartilage thickness within the joint) in the *Au. afarensis* specimen AL 288–1’s hip. The second modeled dynamic translation to ascertain if the sphericity of the hominin hip joint supersedes the need to model dynamic translation of the hip (Manafzadeh & Gatesy 2021), or if the full spectrum of viability is instead only captured via including realistic joint movement (i.e., by modeling all DOFs in a given joint).

If we focus of the results from the first approach, we can confidently state that the AL 288–1 hip joint was not highly congruent thereby dismissing simulations 1–2 based upon too few poses for any type of movement (bipedal or quadrupedal) to be possible. Rather, the likely joint spacing was between ∼1.9–2.5 mm, which is ∼0.68–0.89% of femoral length, although the lower end of this range of spacings does exhibit restricted hip extension necessitated by bipedal walking (Fig. 5) and also of vertical climbing by chimpanzees (Hogervorst & Vereecke 2015; Kozma et al. 2018). In this scenario, AL 288–1 would have to have moved in different ways to achieve the same walking and climbing frameworks as seen in analogous studies if we only look at a static joint center with a thickness towards the ∼1.9 mm range.

The joint becomes more mobile as joint spacing is increased beyond this threshold, although there appears to be a plateau in the production of viable poses, in which greater joint spacing ceases to produce increased viability, whilst the joint start to become disarticulated. This suggests that joint spacing above a ∼2.5 mm threshold is unlikely. Cartilage is powerful to resisting compressive forces, but it is not vascularized. Bone, on the other hand, is highly vascularized and composed of a calcified matrix which promotes strength and resistance to different forms of stress (compressive, tensile, and shear). If thicker cartilage is not permitting greater mobility in AL 288–1 (simulations 4–6), then it is mechanically unlikely—although not entirely infeasible—that thicker cartilage would be present and that the thickness would be above this ∼2.5 mm threshold. However, this is only our assumption and requires further investigation by digitally recreating cartilage and conducting joint reaction force studies (Mathai & Gupta 2019).

Joint spacing is also extremely unlikely to be above 5.3 mm. If joint spacing was increased beyond the 5.3 mm spacing (simulation 6), then the diameter of the femoral head would be displaced/come in line with the acetabular wall, in which ROM is predictably severely limited based upon clinical studies of femoral head displacement (Burroughs et al. 2005; Kessler et al. 2008; Bunn et al. 2014). In fact, the 5.3 mm spacing was already approaching this point of unviability but was included here to provide a full range of extant species’ joint configurations in the AL 288–1’s hip. Further increases in joint spacing (>5.3 mm) would produce a dislocated hip, rather than an articulated one and would be of no functional use.

Using the static translation approach, we are left with two possible scenarios

1. *Au. afarensis* had a relatively more congruent hip joint than modern humans do and thus could not have walked with a modern human-like gait, but rather with more abducted hips. In this scenario, joint spacing would be towards the ∼1.9 mm threshold. This scenario violates the scaling assumption in which smaller bodied animals have less congruent joints than larger animals.
2. The hip joint was more cartilaginous which permitted greater mobility to facilitate a repertoire of movements, which includes the capability to walk bipedally with an extended limb at a range of speeds across multiple substrates (Fig. 5). In this scenario, thickness would be towards the ∼2.5 mm threshold. However, there would be the loss of some flexion (i.e., the blank space in front of the pelvis in simulations 4–6, Fig. 4), in which AL 288–1 could not have positioned the femur in front of the body in the sagittal plane (simulations 4–6; i.e., example “unviable” pose: 50°FE,−5° ABAD,−10° LAR). This pose is equivalent to a standard sitting position whether in a chair or sitting on the floor with the thighs in front of the body for a human. Such flexion is also present in chimpanzees during habitual quadrupedal movement (Eng et al. 2015; Kozma et al. 2018), but missing in AL 288–1 when joint spacing is increased to > 1.9 mm.

Overall, if we only included the first approach in which we systematically increased joint spacing via a static single axis increase, then we would be making broad inferences regarding AL 288–1’s mobility, such as either the inability to extend the hip to move across certain substrates with an erect limb (simulations 1–3; Fig. 5), or the inability to hyper-flex the femur into a sitting/climbing posture (simulations 4–6). Both of these conclusions would drastically change what is already known about the movement capability of this species (Wang et al. 2004; Lovejoy 2005; Nagano et al. 2005; Sellers et al. 2005), despite offering new insights into joint congruency.

Rather, the inclusion of all six DOFs (simulation 9) generated a full spectrum of viable poses. Therefore, the spherical approach which was implemented here to model translational and rotational motion in the hip (a ball and socket joint) is considered accurate and should be used by future studies assessing similar hominin joints. Furthermore, the radii of this sphere should be set to the difference in radii of the fitted shapes—an approach first used by (Demuth et al. 2020). Here, this approach demonstrates that the joint spacing in AL 288–1’s hip was 2.448 mm, whereby the sphere-approach in which the ACS moved to each vertex more closely modeled differing joint spacings within the joint than that of a static approach. In comparison to simulation 4 which modeled a similar (but static) amount of thickness (2.587 mm), numerous poses which were unviable were instead viable, indicating that a single axis translation is not sufficient to capture true mobility. This concludes that six DOFs are required to fully capture the range of poses of the hominin hip joint. If we neglect to include all DOFs, then we would have made erroneous claims regarding osteological functionality of the *Au. afarensis* hip.

It is our assumption that if a larger sphere were used in the six DOF simulation, additional non-realistic joint poses would be inadvertently included, which are non-biological. Such an approach would produce disarticulated joints which, in reality, would be constrained by soft tissues, such as ligaments (Manafzadeh & Padian 2018). A smaller sphere might constrain such poses, but risks not capturing the full spectrum. Rather, future studies should use the 2.448 mm spacing (or within the range of 1.9 to 2.5 mm) with the inclusion of soft tissue constraints to provide a greater insight into AL 288–1’s hip mobility.

If we compare the AL 288–1 six DOF results to the human and chimpanzee specimens, we find non-functional differences in the ROM maps. Therefore, we cannot make any claims regarding possible functional affinities/disparities, nor can we predict how AL 288–1 moved. Rather, we can state that AL 288–1 could osteologically move the femur in the same way as both a human and chimpanzee. Future studies should include soft tissue constraints (Manafzadeh & Padian 2018) to determine how this might restrict each specimen's ROM. Only then could we postulate the functional capability of the AL 288–1 hip, complementing previous studies which have explored hip biomechanics in this specimen (Wang et al. 2004; Nagano et al. 2005; Sellers et al. 2005).

### Palaeobiological implications

Here, we have demonstrated that six DOFs are required for the hominin hip joint, and without such representation of dynamic movement, wrong estimates of function would be made. Whilst previous research has demonstrated the need to include translational movement (Demuth et al. 2020; Richards et al. 2021), full joint sampling has only been conducted rarely in the past (Manafzadeh & Gatesy 2021, 2022). Whilst six DOFs were not required for extant Hominini (the human and chimpanzee), we postulate that this is due to slight differences in the sphericity of the AL 288–1 acetabulum despite both species having highly spherical, contained joints. Humans have a more circular acetabulum than AL 288–1 (Stern & Susman 1983). The human and chimpanzee hips are more contained, whilst the AL 288–1 acetabulum instead has greater cranial expansion and a reduced lateral articular surface, but a greater anterior articular expansion, albeit approaching the modern human range of lunate expansion (MacLatchy 1996). Such differences are evidently influencing predicted mobility by the need to include six DOFs, although these anatomical differences are non-influential if solely modeling osteological viability. Therefore, generic joint shape (i.e., sphericity) cannot underpin modeling assumptions. We must examine morphological differences across the joint surface.

Additionally, the human and chimpanzee specimens were scanned in articulation and thus we were able to precisely locate their respective joint centers. Due to the disarticulated nature of the AL 288–1 specimen, it was not possible to accurately locate the joint center position and, thus, six DOFs are required due to this uncertainty and the presented inadequacies in the four DOF approach to fully capture the joints ROM.

Moving forward, we recommend that translational DOFs are modeled for all joints (in line with Manafzadeh & Gatesy 2021), regardless of any underlying assumptions that they might not be needed (e.g., joint sphericity). Additionally, for extinct species or disarticulated extant species, for which we have no information on joint spacing, the superimposition of fitted shapes (Demuth et al. 2020; Gatesy et al. 2022) might not result in an accurate articulation of the joint nor accurate definition of the rotational joint center (however, different shapes might reduce the magnitude of misalignment; Demuth et al. 2020). To prevent false conclusions on mobility based on inaccurate joint definitions six DOFs are a necessity to accommodate errors introduced in the joint setup. Otherwise, such study might wrongly conclude functional capability or incapability of an extinct species, as would have been the case here if six DOFs had not been included.

### Limitations of the study

Firstly, this study did not model any direct soft tissue constraints, such as ligaments or muscles which act to move the body forward, but which also act to prevent the bones from moving into unviable poses. Moving forward, we would expect the ROM maps produced here to decrease in size with soft tissue constraints (Arnold et al. 2014; Manafzadeh & Padian 2018; Manafzadeh & Gatesy 2021). For example, the current viable poses around the peripheries and those present cranially to the pelvis in all specimens (Figs. 4, 6) are likely to be removed with soft tissue constraints.

Secondly, modeling issues may exist in uncertainties in determining the exact joint center position (Demuth et al. 2020). Differently positioned joint centers may produce slightly different results, as highlighted by the six DOF simulation. Due to the overlap within the results of simulation 9 with those of the human and chimpanzee simulations, we think that the effect of these uncertainties in our case is, however, negligible.

Finally, errors may exist in the reconstruction of the pelvis (Brassey et al. 2018). If other joints in the pelvis (such as the pubic symphysis or sacroiliac joints) were originally modeled with the wrong cartilage spacing, we might find that the pelvis was instead more anteriorly rotated. In that circumstance, we might find that the ROM map may be rotated more anteriorly and so different poses are instead unviable. However, such changes in joints spacing elsewhere in the pelvis would have a minimal effect on the ROM and would likely only change by a few degrees in any given direction, with negligible impact on our results because we only sampled at five degree intervals (Manafzadeh & Padian 2018).

## Conclusion

We tested the articulation and possible osteological ROM of the AL 288–1 hip joint by modeling a static single axis translation to investigate increasing joint spacing, which was considered a proxy for measuring the maximum cartilage thickness. We expanded upon this by including all six DOFs, thereby reflecting true joint movement (Gatesy et al. 2022). Whilst the resultant ROM maps were quite similar, there was a greater spectrum of viability in the six DOF simulation than the other simulations, in which the femur was capable of osteologically moving into a greater range of poses. With this spectrum of poses, AL 288–1 was capable of a repertoire of movements, such as erect bipedalism across a range of substrates at various speeds and vertical climbing. Overall, six DOFs are a requirement for modeling mobility in fossil hominins, otherwise the resultant functionality of a given joint may be wrong.

We conclude that the likely maximum joint spacing/cartilage thickness of AL 288–1’s hip joint was 2.448 mm which is on par with allometric scaling assumptions. Similar estimates were also generated from the single axis translational simulations, despite some implied functional limitations (simulation 4).

## Supplementary Material

obac031_Supplemental_Files

## Data Availability

All data used in this study was obtained from previous studies.
