# Supplementary material for: Reconstructing Articular Cartilage in the Australopithecus afarensis Hip Joint and the Need for Modeling Six Degrees of Freedom
Source: Integr Org Biol. 2022 Jul 28;4(1):obac031. doi: 10.1093/iob/obac031 (PMC9428927; doi:10.1093/iob/obac031)
Supplement: obac031_Supplemental_Files [file obac031_supplemental_files.zip › SI_1_revised.docx]

**Supplementary Information 1.**

**Reconstructing articular cartilage in the *Australopithecus afarensis* hip joint and the need for modelling six degrees of freedom**

Authors: Ashleigh L. A. Wiseman^1,2^. Oliver E. Demuth^3,4^, Emma Pomeroy^5^, Isabelle De Groote^6^

^1^McDonald Institute for Archaeological Research, University of Cambridge, UK. ^2^Research Centre in Evolutionary Anthropology and Paleoecology, Liverpool John Moores University, Liverpool, UK. ^3^Department of Earth Sciences, University of Cambridge, UK. ^4^Structure and Motion Laboratory, Royal Veterinary College, UK. ^5^Department of Archaeology, University of Cambridge, UK. ^6^Department of Archaeology, Ghent University, Ghent, Belgium.

Author for correspondence: Ashleigh L. A. Wiseman. Email: [alw96@cam.ac.uk](mailto:alw96@cam.ac.uk).

**Experimental data capture from modern humans walking at varied speeds on different substrates (for full details on trackway construction see (Wiseman, 2019; Wiseman & De Groote, 2021)).**

Adult participants (20 females and 20 males; 19 - 40 years old) were recruited to walk across three different substrates of differing hydrology. Two trackways were constructed that were filled with fine-grained homogenous sand composed of rounded to sub-angular particles measuring ~0.06-0.7 mm in diameter, with a standardised depth of 44 mm. Two different water contents were chosen for each trackway: a low-water content (6-8%) and a high-water content (10-12%), following protocol developed by (Raichlen et al., 2010). Similar sand/hydration levels have been used in other studies (Wiseman et al., 2020) and are reported to have a similar consistency to the Laetoli substrates (Crompton et al., 2012) which were composed of nephelinite volcanic ash (Leakey, 1979). An additional trackway (the ‘controlled walk’) was included which consisted of the hard ground (laboratory floor) to validate habitual movement (i.e., walking on soft, deformable sands in likely to alter how a person walks, and so to control that joint angles were consistently reported, results were compared to the controlled walk). Each trackway measured 12 m long by 0.6 m wide.

Participants were asked to walk with at a self-selected walking speed, a fast walk and then a jog across each of the constructed substrates. If speed varied by >0.2 m/s (speed discrepancy arbitrarily determined during the experiments based upon realistically capturing repeatable data) on any substrate, the trial was discarded and recaptured. All motion trials were repeated five times (Levine et al., 2012). Between each individual trial the experimental trackways were flattened and levelled using a screed to ensure that all steps were conducted on a flat surface.

A 14-camera optoelectronic 3D motion capture system (250 Hz, Oqus Cameras, Qualysis AB, Gothenburg, Sweden) was employed to quantify movement across each substrate via reflective marker-set. All participants agreed to wear minimal clothing for adequate marker placement directly onto bony anatomical landmarks according to the Liverpool John Moores University Lower Limb and Trunk Model, allowing six degrees of freedom for the functional assessment of the hip, knee and ankle joints (Vanrenterghem et al., 2010).

Visual3D (v.5.02.30 C-Motion Inc., Germantown, USA) was used for the creation of a biomechanical model to estimate kinematics. Rigid segments were defined using the Visual3D protocol based upon each of the static trials. Data was interpolated to fill any remaining gaps and a Butterworth low-pass filter of 6.0 Hz was used to smooth marker trajectories. All gait cycles belonging to each participant from each motion on a particular substrate (n≈45 gait cycles per condition; nine strides per each 12m trial extracted) were averaged to provide one mean (with range) gait cycle. Hip joint angles from heel strike to heel strike of the same foot (the start and end of one complete gait cycle) in the sagittal plane were calculated as the angle between the pelvis and thigh segments.

Crompton, R. H., Pataky, T. C., Savage, R., D'Aout, K., Bennett, M. R., Day, M. H., Bates, K., Morse, S., & Sellers, W. I. (2012). Human-like external function of the foot, and fully upright gait, confirmed in the 3.66 million year old Laetoli hominin footprints by topographic statistics, experimental footprint-formation and computer simulation. *Journal of Royal Society Interface*, *9*(69), 707-719. <https://doi.org/10.1098/rsif.2011.0258>

Leakey, M. D., Hay, R. L. (1979). Pliocene footprints in the Laetolil Beds at Laetoli, northern Tanzania. *Nature*, *278*, 317-323.

Raichlen, D. A., Gordon, A. D., Harcourt-Smith, W. E., Foster, A. D., & Haas, W. R. (2010). Laetoli footprints preserve earliest direct evidence of human-like bipedal biomechanics. *PLoS One*, *5*(3), e9769. <https://doi.org/10.1371/journal.pone.0009769>

Vanrenterghem, J., Gormley, D., Robinson, M., & Lees, A. (2010). Solutions for representing the whole-body centre of mass in side cutting manoeuvres based on data that is typically available for lower limb kinematics. *Gait Posture*, *31*(4), 517-521. <https://doi.org/10.1016/j.gaitpost.2010.02.014>

Wiseman, A. L., Bezombes, F., Moore, A. J., & De Groote, I. (2020). Non-invasive methods: The applicability of unmanned aerial vehicle (UAV) technology for recording fossilised footprints. *Digital Applications in Archaeology and Cultural Heritage*, *16*. <https://doi.org/10.1016/j.daach.2020.e00137>

Wiseman, A. L. A. (2019). *Improving Recording and Interpretation of Fossil Tracks* [PhD Thesis, Liverpool John Moores University].

Wiseman, A. L. A., & De Groote, I. (2021). One size fits all? Stature estimation from footprints and the effect of substrate and speed on footprint creation. *The Anatomical Record (Hoboken)*, 1-9. <https://doi.org/10.1002/ar.24833>
