# Supplementary material for: Reconstructing Articular Cartilage in the Australopithecus afarensis Hip Joint and the Need for Modeling Six Degrees of Freedom
Source: Integr Org Biol. 2022 Jul 28;4(1):obac031. doi: 10.1093/iob/obac031 (PMC9428927; doi:10.1093/iob/obac031)
Supplement: obac031_Supplemental_Files [file obac031_supplemental_files.zip › SI_2.docx]

**Supplementary Information 2**

***Computing translational simulations***

Here we provide a MEL (*Maya Embedded Language*) script which computes the translational simulation, adapted from (Manafzadeh & Gatesy, 2021) and implemented in Maya 2022 via the *Expression Editor*. Here, a sphere was used composed of 32 faces (axis division of 8; height division of 4 – created prior to running the script) in which the ACS location moved to each vertex and the joint was then rotated around a total of 197,173 possible poses for all simulations.

int $viable = 0;

*setKeyframe* -at viable -v $viable Hip_Joint;

//loop through translation combinations and mark pose as viable (1) as soon as any translation combination results in a boolean mesh surface area of 0

int $i;

int $j;

int $k;

vector $VtxPos;

int $numbVtx[] = `*polyEvaluate* -v "Acetab_trans_Sphere"`;

for($l=0; $l < $numbVtx[0]+1; $l++)

{

if ($l == $numbVtx[0]+1)

{

$VtxPos = `*xform* -q -t -os ("Acetab_trans_Sphere")`;

}

else

{

$VtxPos = `*xform* -q -t -os ("Acetab_trans_Sphere"+".vtx["+$l+"]")`;

}

if ($viable==0)

{

setAttr "Hip_acs.translateX" ($VtxPos.x);

setAttr "Hip_acs.translateY" ($VtxPos.y);

setAttr "Hip_acs.translateZ" ($VtxPos.z);

float $area[] = `*polyEvaluate* -area boo`;

if ($area[0]>0)

{

$viable = 0;

}

else

{

$viable = 1;

*setKeyframe* -at viable -v $viable Hip_Joint;

}

}

}

***Visualising the results: creating the ROM map***

Below, we provide the MEL script for creating the solid ROM spheres, as shown in Figure 4. This script creates a polysphere which keys the translation of the knee ACS position. Once this is keyed, (1) export the keyed translations into a CSV file, (2) import into MATLAB, (3) use the translations to create a solid shape of the keyed points (script provided below), and then (4) export from MATLAB and reimport back into Maya.

int $nFrames = 63821; //set according to viable array/frame numbers

string $kneeACS = "Knee_acs"; //name according to knee ACS name

float $SphereRadius = 2.5; //set according to preference

/********************************************************************/

int $i;

int $frame =1;

*currentTime* -edit 1;

string $sn = "Pos_Sphere";

*sphere* -p 0 0 0 -ax 0 0 1 -ssw 0 -esw 360 -r $SphereRadius -d 3 -ut 0 -tol 0.01 -s 4 -nsp 2 -ch 1;

*rename* "nurbsSphere1" $sn;

for ($i = 1; $i < ($nFrames + 1); $i = $i + 1)

{

vector $KneePos = `*xform* -q -t -ws $kneeACS`;

*setKeyframe* -at translateX -v ($KneePos.x) -t $frame $sn;

*setKeyframe* -at translateY -v ($KneePos.y) -t $frame $sn;

*setKeyframe* -at translateZ -v ($KneePos.z) -t $frame $sn;

$frame = $frame + 1;

*currentTime* -edit $frame;

}

//then export the keyed frames of the polysphere into MATLAB

To create the solid ROM map shape in MATLAB, use the following in MATLAB. Reimport the stl into Maya. The below script has been tested and works in MATLAB versions 2020a and 2021a.

%% ===== Load data into MATLAB =====

file = 'ForROMMAP_OverlaidShapes.csv'; %change to name of CSV file

ROMenvelope = readtable(file);

EnvelopeArray = table2array(ROMenvelope);

EnvelopeArray(:,1) =[];

EnvelopeDelaunay = delaunayTriangulation(EnvelopeArray(:,1),EnvelopeArray(:,2),EnvelopeArray(:,3));

[K,v] = convexHull(EnvelopeDelaunay);

TR = triangulation(K,EnvelopeDelaunay.Points(:,1), EnvelopeDelaunay.Points(:,2), EnvelopeDelaunay.Points(:,3));

numTriangles = size(TR,1);

Areas = zeros(numTriangles,1);

for i=1:numTriangles

vertex1=[TR.Points(TR.ConnectivityList(i,1),1),TR.Points(TR.ConnectivityList(i,1),2),TR.Points(TR.ConnectivityList(i,1),3)];

vertex2=[TR.Points(TR.ConnectivityList(i,2),1),TR.Points(TR.ConnectivityList(i,2),2),TR.Points(TR.ConnectivityList(i,2),3)];

vertex3=[TR.Points(TR.ConnectivityList(i,3),1),TR.Points(TR.ConnectivityList(i,3),2),TR.Points(TR.ConnectivityList(i,3),3)];

Areas(i) = 0.5*norm(cross(vertex2-vertex1, vertex3-vertex1));

end

%%

test = K;

TF1 = Areas(:,1)>75; %change me depending on how dense the connectivity should be; subjective.

trisurf(test,EnvelopeDelaunay.Points(:,1), EnvelopeDelaunay.Points(:,2), EnvelopeDelaunay.Points(:,3));

TR2 =triangulation(test,EnvelopeDelaunay.Points(:,1), EnvelopeDelaunay.Points(:,2), EnvelopeDelaunay.Points(:,3));

stlwrite(TR2, 'ROM_envelope.stl'); %import this stl back into Maya to visualise the ROM map

Manafzadeh, A. R., & Gatesy, S. M. (2021). Paleobiological reconstructions of articular function require all six degrees of freedom. *Journal of Anatomy*, *239*(6), 1516-1524. <https://doi.org/10.1111/joa.13513>
