# Supplementary material for: Reconstructing Articular Cartilage in the Australopithecus afarensis Hip Joint and the Need for Modeling Six Degrees of Freedom
Source: Integr Org Biol. 2022 Jul 28;4(1):obac031. doi: 10.1093/iob/obac031 (PMC9428927; doi:10.1093/iob/obac031)
Supplement: obac031_Supplemental_Files [file obac031_supplemental_files.zip › SI_3_revised.docx]

**Supplementary Information 3**

**Reconstructing articular cartilage in the *Australopithecus afarensis* hip joint and the need for modelling six degrees of freedom**

Authors: Ashleigh L. A. Wiseman^1,2^. Oliver E. Demuth^3,4^, Emma Pomeroy^5^, Isabelle De Groote^6^

^1^McDonald Institute for Archaeological Research, University of Cambridge, UK. ^2^Research Centre in Evolutionary Anthropology and Paleoecology, Liverpool John Moores University, Liverpool, UK. ^3^Department of Earth Sciences, University of Cambridge, UK. ^4^Structure and Motion Laboratory, Royal Veterinary College, UK. ^5^Department of Archaeology, University of Cambridge, UK. ^6^Department of Archaeology, Ghent University, Ghent, Belgium.

Author for correspondence: Ashleigh L. A. Wiseman. Email: alw96@cam.ac.uk.

Here, we report the individual alpha shapes from the AL 288-1 simulations (simulations 1-6; 9) to complement Figure 4 (Figure S1). Minute shape differences can be clearly visualised in the below figure. For example, simulation 1 (0.764 mm cartilage thickness) has many floating ‘islands’ and the loss of most joint mobility. In this scenario, AL 288-1 would have been unlikely to walk bipedally, quadrupedally or even climb. Thus, simulation 1 can be discarded as non-biological.

**
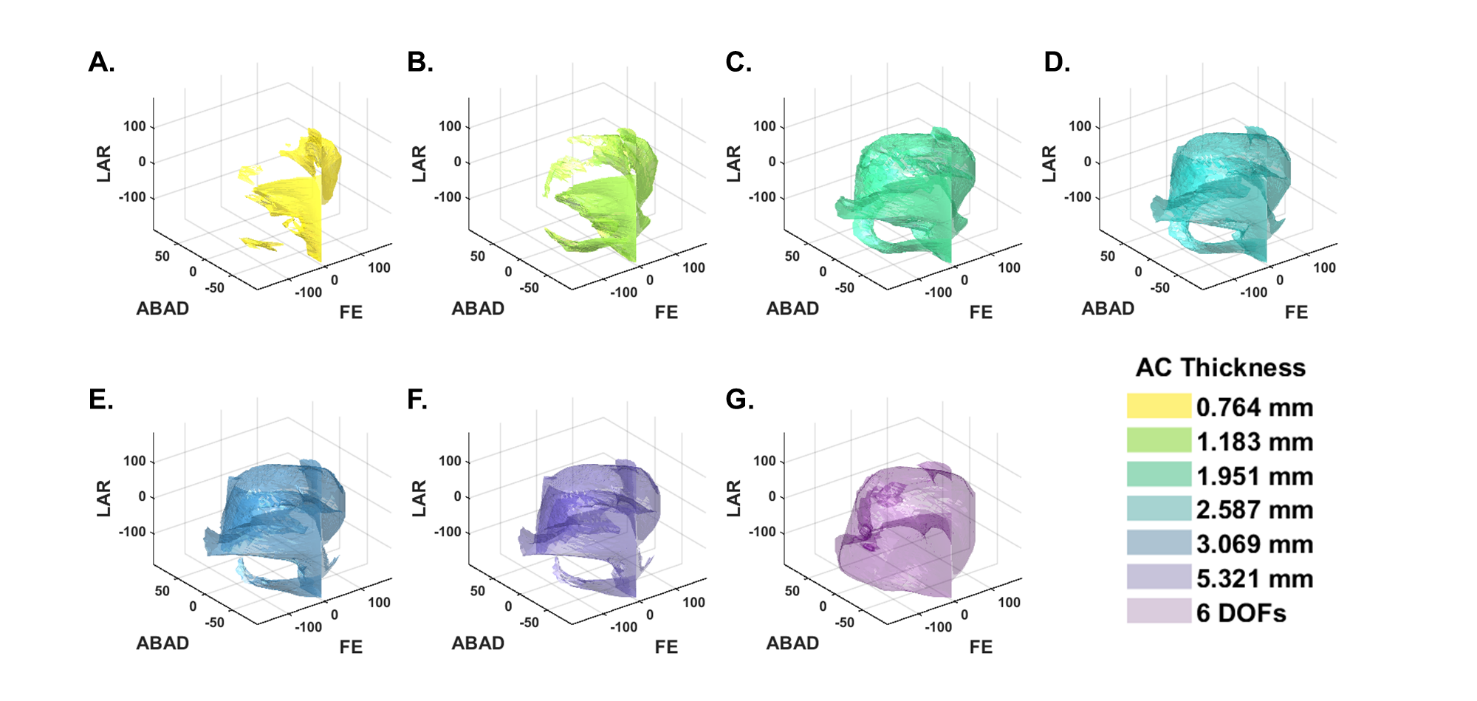
**

**Figure S1.** The ROM maps for AL 288-1 with cartilage thickness of (A) 0.764 mm – simulation 1, (B) 1.183 mm – simulation 2, (C) 1.951 mm – simulation 3, (D) 2.587 mm – simulation 4, (E) 3.069 mm – simulation 5, (F) 5.321 mm – simulation 6, and (G) the six degree of freedom (DOF) approach – simulation 9.

We also report the results of the alpha shapes from the six DOF simulation overlaid with that of the four DOF simulation (static Y-axis translation approach) (Figure S2). The predicted cartilage thickness in our study was 2.448 mm (simulation 9), which was close to the value which was predicted from the static Y-axis translation simulations (2.587 mm; simulation 4), although the latter had functional limitations. Below we directly compare the alpha shapes from simulations 4 and 9. We find that the four DOF approach (with a static translational offset) has a reduced amount of viable joint configurations, of which there are multiple associated functional limitations (see text). On the other hand, the results of the six DOF simulation produces an alpha shape which fully envelopes the shape space, with no predicted functional restrictions and which resembles that of both the human and chimpanzee. Consequently, six DOFs are a requirement for ROM mapping studies.

**
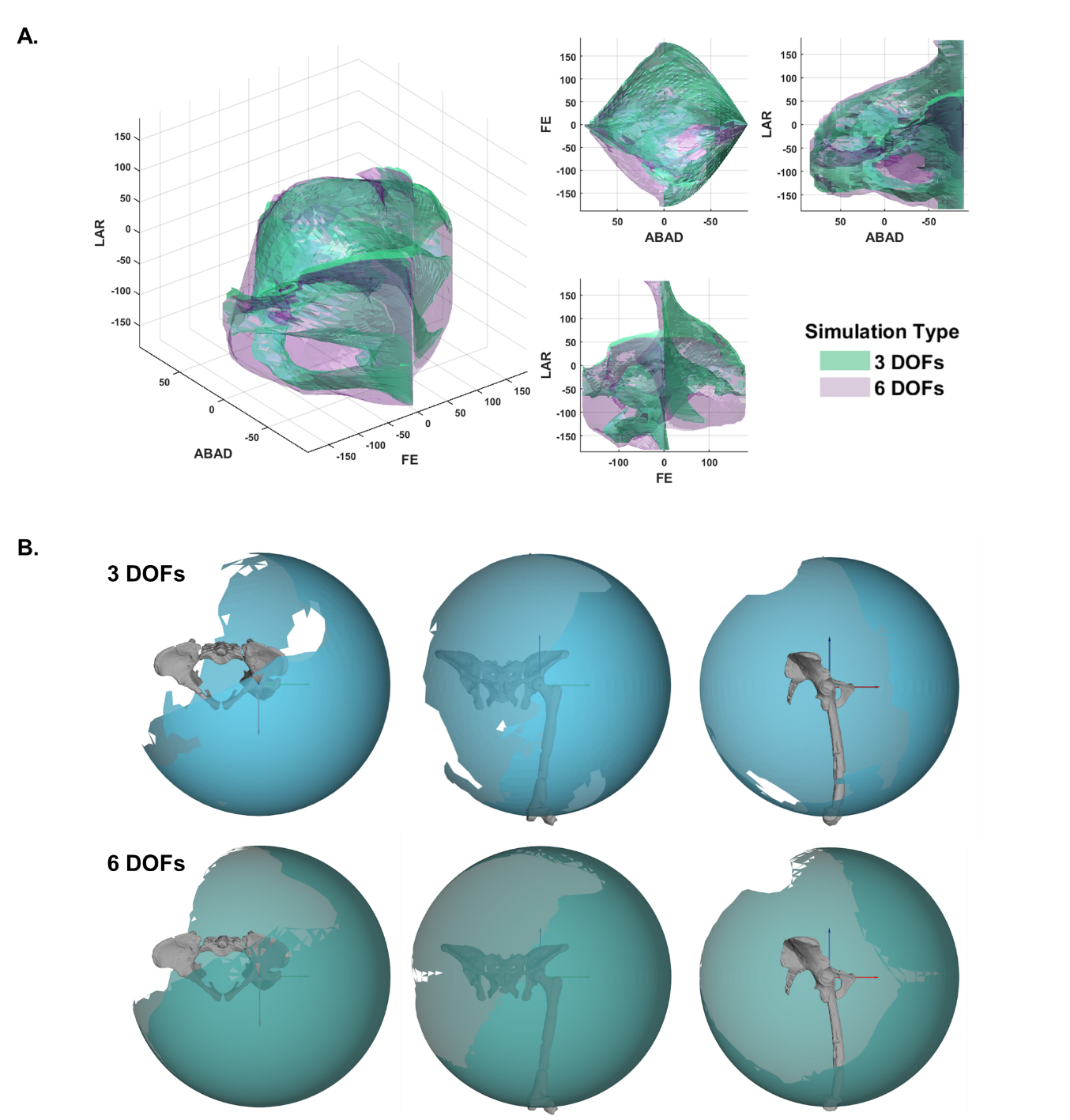
**

**Figure S2.** (A) 3D plots illustrating the shape differences between each of the alpha shapes associated with the two modelling approaches used in this study (simulation 4 and simulation 9), representing the *Au. Afarensis* ROM mapping. (B) The ROM maps for AL 288-1 from the four DOF simulation (static translation approach; simulation 4) and the six DOF simulation.
